# A novel cuproptosis-related lncRNAs signature predicts prognostic and immune of bladder urothelial carcinoma

**DOI:** 10.3389/fgene.2023.1148430

**Published:** 2023-03-31

**Authors:** Zheng Zhou, Yusong Zhou, Wei Liu, Jing Dai

**Affiliations:** ^1^ Department of Otolaryngology Head and Neck, The Third Xiangya Hospital, Central South University, Changsha, China; ^2^ Department of Pharmacy, The Third Xiangya Hospital, Central South University, Changsha, China; ^3^ Department of Gastrointestinal Surgery, The Third Xiangya Hospital, Central South University, Changsha, China

**Keywords:** lncRNA, cuproptosis, BLCA, prognostic, immune infiltrates, immune checkpoints

## Abstract

Bladder Urothelial Carcinoma (BLCA) remains the most common urinary system tumor, and its prognosis is poor. Cuproptosis is a recently discovered novel cell death involved in the development of tumor cells. However, the use of cuproptosis to predict the prognosis and immunity of Bladder Urothelial Carcinoma remains largely unclear, and this study was designed to verify cuproptosis-related long non-coding RNAs (lncRNAs) to estimate the prognosis and immunity of Bladder Urothelial Carcinoma. In our study, we first defined the expression of cuproptosis-related genes (CRGs) in BLCA, and 10 CRGs were up- or downregulated. We then constructed a co-expression network of cuproptosis-related mRNA and long non-coding RNAs using RNA sequence data from The Cancer Genome Atlas Bladder Urothelial Carcinoma (TCGA-BLCA), clinical features and mutation data from BLCA patients to obtain long non-coding RNAs by Pearson analysis. Afterward, univariate and multivariate COX analysis identified 21 long non-coding RNAs as independent prognostic factors and used these long non-coding RNAs to construct a prognostic model. Then, survival analysis, principal component analysis (PCA), immunoassay, and comparison of tumor mutation frequencies were performed to verify the accuracy of the constructed model, and GO and KEGG functional enrichment analysis was used to verify further whether cuproptosis-related long non-coding RNAs were associated with biological pathways. The results showed that the model constructed with cuproptosis-related long non-coding RNAs could effectively evaluate the prognosis of BLCA, and these long non-coding RNAs were involved in numerous biological pathways. Finally, we performed immune infiltration, immune checkpoint and drug sensitivity analyses on four genes (TTN, ARID1A, KDM6A, RB1) that were highly mutated in the high-risk group to evaluate the immune association of risk genes with BLCA. In conclusion, the cuproptosis-related lncRNA markers constructed in this study have evaluation value for prognosis and immunity in BLCA, which can provide a certain reference for the treatment and immunity of BLCA.

## 1 Introduction

Bladder urothelial carcinoma (BLCA) is a urological disease with high morbidity and mortality, and BLCA ranks seventh among male cancers and 17th among female cancers worldwide. Each year, approximately 75% of newly diagnosed BLCA are non-invasive, with approximately 110,500 males and 70,000 females. 38,200 patients in the EU and 17,000 in the US died due to BLCA (Burger et al., 2013). The incidence of BLCA is high among patients over 50 years old. The average age of diagnosis is 69 years for men and 71 years for women (Solomon and Hansel, 2015). Moreover, it has been confirmed that decarboxylase protein complexes are associated with BLCA susceptibility, while decarboxylase protein complexes are potential targets for drug therapy (Cheng et al., 2016). However, there is still a lack of specific biomarkers and research methods for the prognosis and immunity of BLCA patients.

Tsvetkov et al. discovered that cuproptosis is a novel programmed cell death (Tsvetkov et al., 2022). Excessive copper exposure can cause mitochondrial damage in cells leading to different forms of cell death (Kahlson and Dixon, 2022). Whereas copper is one of the indispensable materials in biological processes, the processes involved include mitochondrial respiration, iron absorption, antioxidant/detoxification, and so on (Ruiz et al., 2021). Increasing evidence suggests that copper is involved in the development and progression of tumor cells (Hu et al., 2022; Oliveri, 2022). However, unlike apoptosis, pyroptosis, necroptosis and ferroptosis, research on cuproptosis in cancer prognosis and immunity, including BLCA, remains limited. Recently, many researchers have confirmed that long non-coding RNAs (lncRNAs) are closely related to tumorigenesis, cardiovascular disease, neurological disease, kidney disease and other diseases, and are widely involved in metabolic, immune and other key physiological processes. Studies have found that lncRNAs can be used as biomarkers or intervention targets and have provided a new basis for diagnosing, treating, and prognosis of diseases (Chen et al., 2021). Research has found significant roles of lncRNAs in regulating epithelial-to-mesenchymal transition (EMT), metastasis and treatment response in urological cancers (Hashemi et al., 2022).

Although recent studies have also investigated cuproptosis-related lncRNAs in the prognosis and immunity of BLCA (Zhang et al., 2022), research in this area needs to be continuously explored to improve the treatment outcome and prognosis of BLCA patients. Therefore, in our research, we built a prognostic model of cuproptosis-related lncRNAs in BLCA and assessed the model accuracy while studying the immune infiltration, drug sensitivity and immune checkpoint analysis of highly mutated genes in the high-risk group to provide a scientifically useful basis for further studies.

## 2 Methods and materials

### 2.1 Data sets and preprocessing

On 23 June 2022, 406 BLCA patients were obtained by the Cancer Genome Atlas (TCGA) database, and Supplementary Table S1 showed the clinical information of BLCA patients. Meanwhile, we obtained datasets and sample extraction, including 431 RNA sequencing data (RNA-seq), 412 clinical features, and 415 mutation data, from BLCA patients from the Cancer Genome Atlas-Bladder Cancer Database (TCGA-BLCA, https://portal.gdc.cancer.gov/). R (version 4.2.1) and R Bioconductor packages were used to perform the data analysis.

### 2.2 Expression of CRGs in BLCA

From previous studies (Polishchuk et al., 2019; Aubert et al., 2020; Dong et al., 2021; Ren et al., 2021; Bian et al., 2022; Chen, 2022; Kahlson and Dixon, 2022; Tsvetkov et al., 2022), we obtained a total of 19 CRGs, and the detailed gene names are shown in Supplementary Table S2. Differential expression of CRGs in BLCA and normal adjacent tissues is shown by the R software package ''ggplot2''.

### 2.3 Identify CupRLSig to predict prognosis in patients with BLCA

The absolute value of the Pearson association coefficient (>0.4) and *p* < 0.05 were regarded as thresholds for establishing a cuproptosis-related mRNA-lncRNA co-expression network to verify lncRNAs’ relevance in cuproptosis. By the R software package ''ggalluvial'', the network was visualized using a Sankey diagram. A heatmap of the cuproptosis-related lncRNAs and CRGs was drawn using BiocManager ''limma'', ''tidyverse'', ''ggplot2″ and ''ggExtra'' packages. Subsequently, the entire TCGA-BLCA samples were randomly divided into training and test groups; we used a univariate Cox regression analysis to verify whether these lncRNAs were associated with patient prognosis in the training group. We also used the lasso regression analysis to avoid overfitting and remove closely related genes. Subsequently, the above lncRNAs were used to build multiple Cox regression models and verify association coefficients. We formulated the resulting model risk score as follows: risk score = $\sum_{i=1}^{n}\left(explncRNAi\times coef-lncRNAi\right)$ . This prognostic lncRNA signature was called CupRLSig. We calculated the risk scores for each patient from the all, training, and test BLCA groups, and based on the median risk score of the training group, BLCA samples from all three groups were classified into two categories: high-risk and low-risk groups. Then, the Kaplan-Meier curves, hazard curves, survival status, and heatmap analyses were used to verify whether the CupRLSig model obviously differentiated the patients in high- and low-risk groups. Meanwhile, the progression-free survival (PFS), concordance index (C-index), independent prognostic analysis, and receiver operating characteristic (ROC) curves were also used to verify model accuracy. By using the R package ''pheatmap'', clinicopathological variables were visualized in the high- and low-risk groups from the entire TCGA-BLCA sample set; using principal component analysis (PCA), we assessed the distribution of patients with different risk scores, and using the R package ''scatterplot3d'' to visualization. Eventually, using various pathological parameters, stratified analyses were performed to verify whether the model was significantly associated with other clinical parameters between the high-risk and low-risk groups.

### 2.4 Building nomograms and calibration curves

Then, according to a combination of risk scores and other clinicopathological data, we used the R software packages ''rms'' and ''regplot'' to build the nomograms, aimed to obtain the survival of BLCA patients at 1, 3, and 5 years. Using the calibration curve, we assessed if the predicted survival agreed with actual survival and selected patients to validate the predictive effectiveness of the nomogram at random.

### 2.5 Independent prognostic analysis and principal component analysis

The independent prognostic analysis graph was drawn using the R package ''survival'' and the model validation of the principal component analysis graph clinical grouping was drawn using the BiocManager ''limma'' and ''scatterplot3d'' packages based on the TCGA transcriptional sample database and clinical sample database, and the model validation curve of the clinical high and low-risk grouping was drawn using the ''survival'' and ''survniner'' packages.

### 2.6 Functional enrichment analysis of cuproptosis-related lncRNAs

By http://vip.sangerbox.com/home.html, we performed Gene Ontology (GO) and Kyoto Encyclopedia of Genes and Genomes (KEGG) functional enrichment analysis on differentially expressed genes and lncRNAs.

### 2.7 Immune-related function analysis and gene mutation frequency comparison

BLCA immune-related functional graphs were drawn using the BiocManager ''limma'', BiocManager ''GSVA″, BiocManager ''ABase'', ''pheatmap'', and ''reshape2″ packages and gene mutation frequency waterfalls were drawn using the BiocManager ''maftools'' package in the high-risk and low-risk CupRLSig groups.

### 2.8 Tumor mutation burden and survival analysis

Using the R software package ''maftools'', somatic non-synonymous point mutations were counted and visualized in each sample. Furthermore, we compared tumor mutation burden (TMB) between low- and high-risk groups and plotted the risk score survival curve for TMB.

### 2.9 High-mutation genes are associated with tumor immune infiltration, drug sensitivity and immune checkpoint analysis in BLCA

We then used the Tumor Immunity Estimation Resource (TIMER, https://cistrome.shinyapps.io/timer/) to analyze the association between highly mutated genes (TTN, ARID1A, KDM6A, RB1) in high-risk groups and immune infiltration, a portal that comprehensively analyzes tumor-infiltrating immune cells. After that, from (http://bioinfo.life.hust.edu.cn/GSCA/#/drug), these four highly mutated genes and CTRP drug sensitivity were analyzed. Finally, we performed an immune checkpoint analysis of BLCA patients *via* the TCGA database. Correlation analysis was performed on three significantly different immune checkpoint genes (HAVCR2, PDCD1LG2, SIGLEC15) and four highly mutated genes by the "pheatmap" package. The gene expression comparison of the three immune checkpoint genes in the high and low risk groups was carried out by the "ggplot2" package.

## 3 Results

### 3.1 Expression of CRGs in BLCA

**FIGURE 1:**
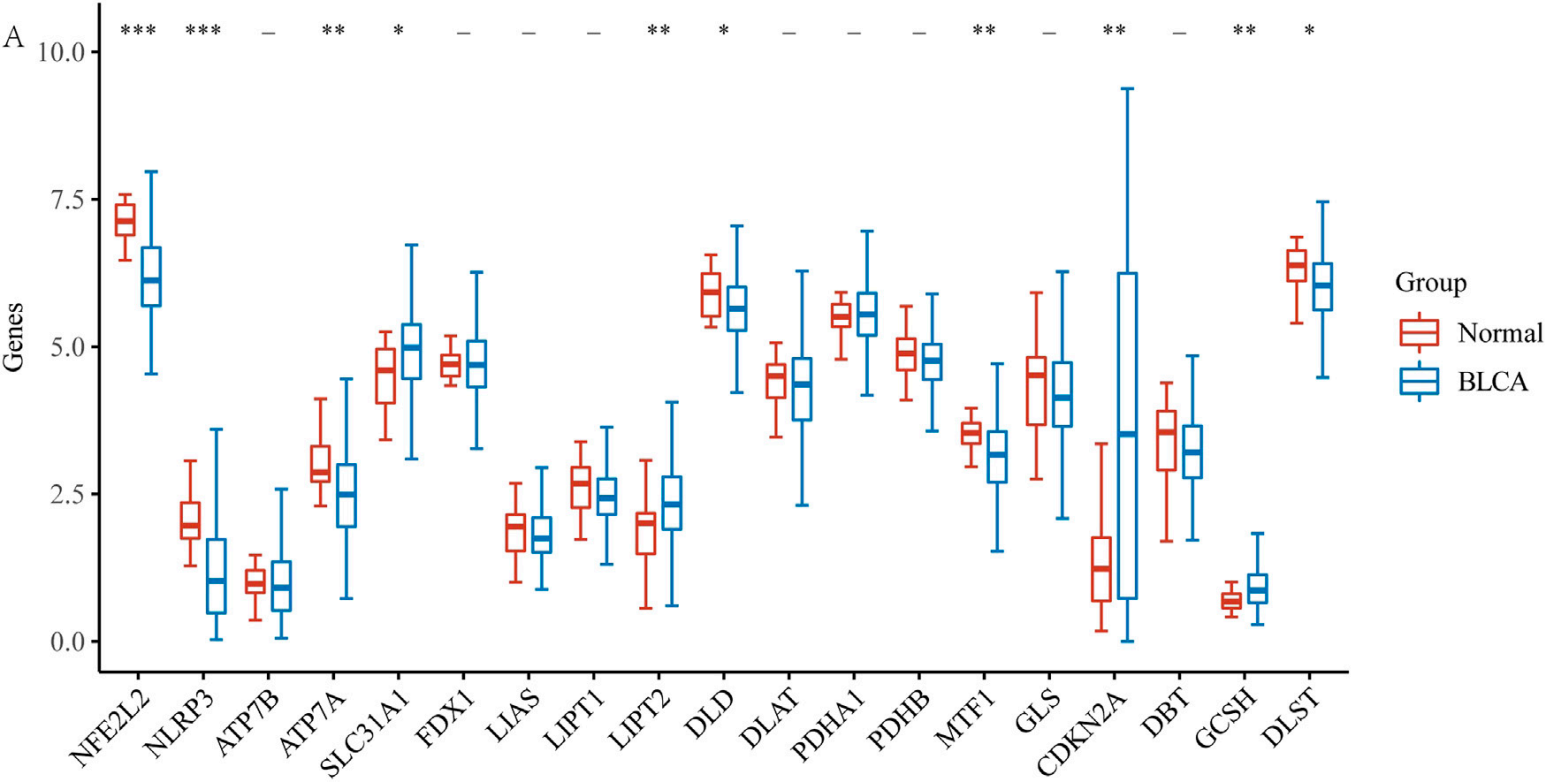
The expression of CRGs in BLCA (*p < 0.05, **p < 0.01,***p < 0.001, asterisks (*) stand for significance levels.).

First, using the BLCA dataset, we explored the expression of 19 CRGs in BLCA and normal adjacent tissues. Results showed increased expression of four genes and decreased expression of six genes in BLCA (Figure 1A). To be more exact, SLC31A1, LIPT2, CDKN2A, and GCSH expression increased, while NFE2L2, NLRP3, ATP7A, DLD, MTF1, and DLST expression decreased in tumor tissues.

### 3.2 Construction of CupRLSig model

**FIGURE 2:**
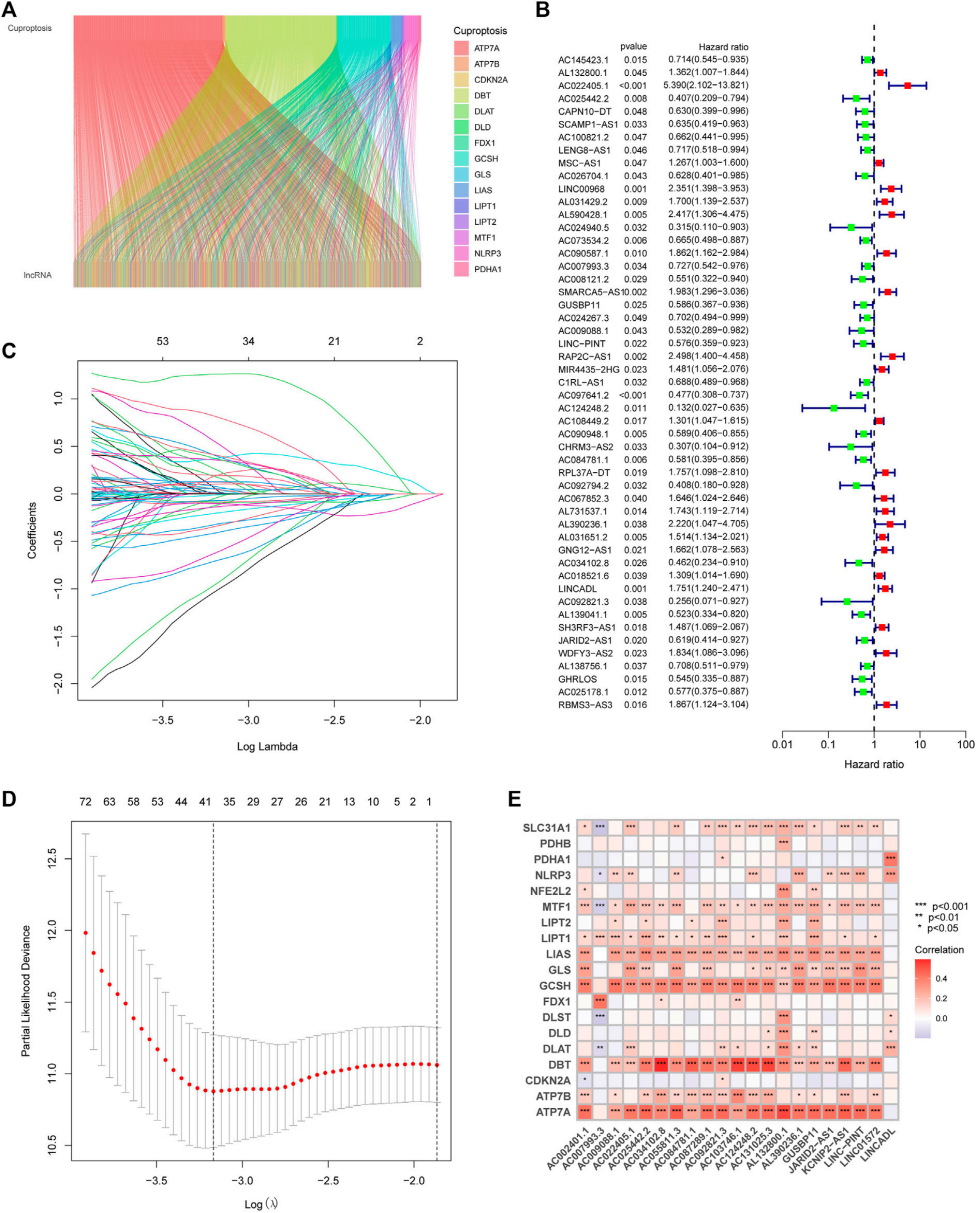
Construction of the CupRLSig model **(A)** The Sankey diagram shows the relevance between cuproptosis-related lncRNAs and cuproptosis-related genes **(B)** Forest plot showed different lncRNAs for high and low risk, with red representing high-risk lncRNAs and green representing low-risk lncRNAs **(C)** The 10-fold cross-validation of variable selection in the least absolute shrinkage and selection operator (LASSO) algorithm **(D)** Distribution of the LASSO coefficients of cuproptosis-related lncRNAs **(E)** A heatmap shows the association between the lncRNAs and 19 CRGs.

**FIGURE 3:**
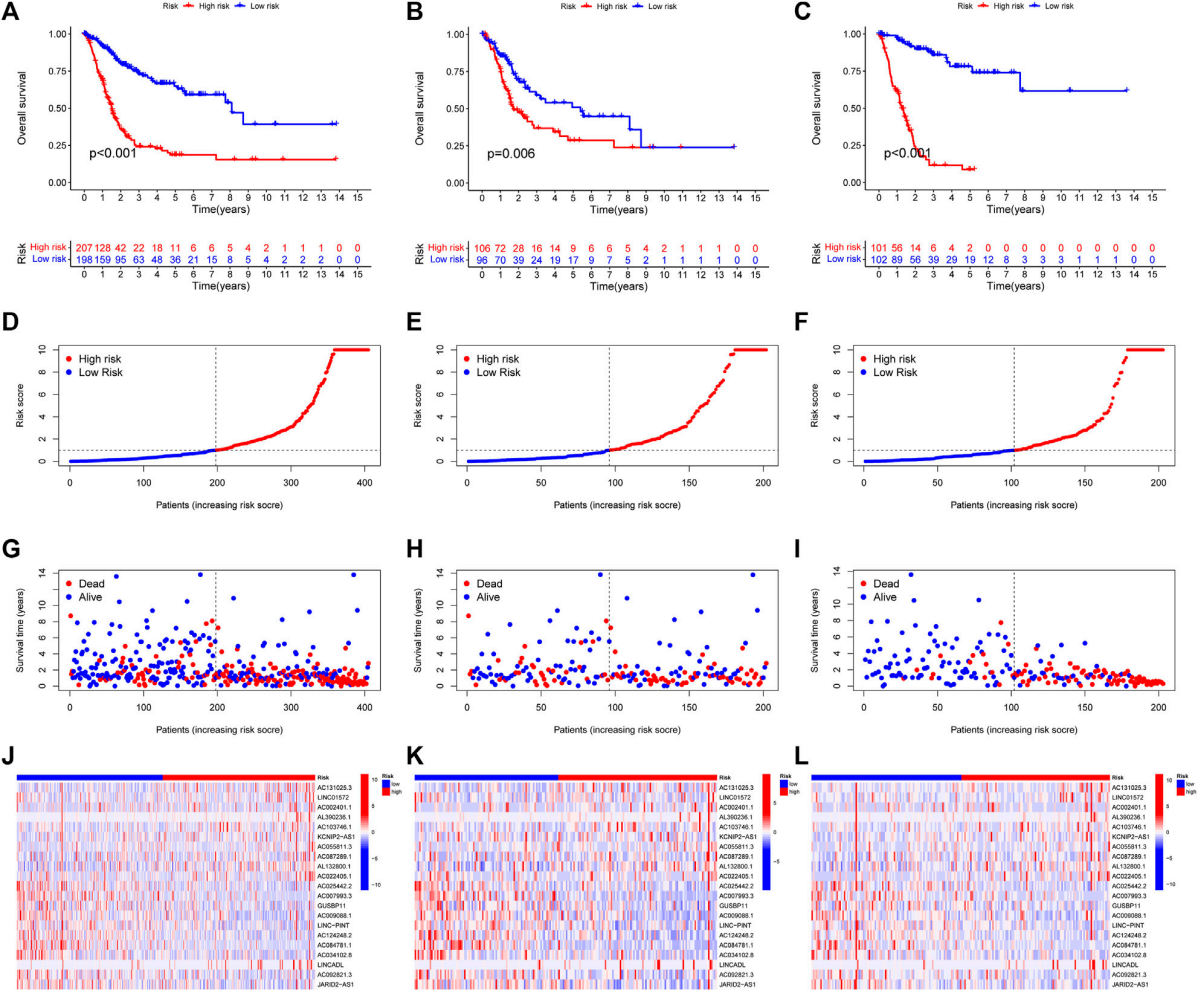
CoInternal validation for CupRLSig model overall survival determination for the all, train and test TCGA-BLCA groups. Prognosis of the risk model in different groups. Kaplan–Meier survival curves of overall survival of BLCA patients **(A–C)**, The distribution of overall survival risk scores **(D–F)**, survival time and survival status **(G–I)**, heatmaps of 21 lncRNA expressions **(J–L)**. Red and blue dots indicate death and survival, respectively.

With the |R| >0.4 and *p* < 0.001 as the analysis criteria, 1,426 cuproptosis-related lncRNAs were identified from 16,876 lncRNAs. Pearson association analysis determined the cuproptosis-related lncRNAs associated with 15 CRGs (Figure 2A). According to the optimal penalty parameter (λ) value, in the training group, univariate Cox regression analysis identified 51 lncRNAs, including 29 low-risk lncRNAs and 22 high-risk lncRNAs (Figure 2B). Lasso Cox regression analysis identified 40 cuproptosis-related lncRNAs, we identified trajectory changes in regression coefficients of lncRNAs and cross-validation results of model construction (Figures 2C, D). Multivariate COX analysis identified 21 lncRNAs as independent prognostic factors. The association between the 21 lncRNAs and 19 CRGs was shown in Figure 2E. The risk score of each sample was then calculated based on the expression of 21 lncRNAs. Risk score = (1.6195×AC131025.3 expression) + (0.0.8528×LINC01572 expression) + (1.8513×AC002401.1 expression) + …+ (−0.4657×JARID2-AS1 expression), see Supplementary Table S3 for details. Then, we used this formula to calculate the risk score for each patient. Based on the training group’s median risk score (Median risk score is 1), we divided the patients into two risk groups. Eventually, in the three training, testing and all group, 106, 101 and 207 patients were divided into the high-risk group; at the same time, 96, 102, and 198 patients were divided into low-risk groups (Figures 3A–C). Kaplan-Meier analysis showed that the OS of the high-risk group was significantly shorter than that of the low-risk group in the two data sets (Figures 3A–C). Figures 3D–I expands on the individual patient risk score and survival statistics. Increased mortality with increasing risk score. Figure 3J-L details the expression status of 21 lncRNAs in each group.

### 3.3 Evaluate the accuracy of the CupRLSig model

**FIGURE 4:**
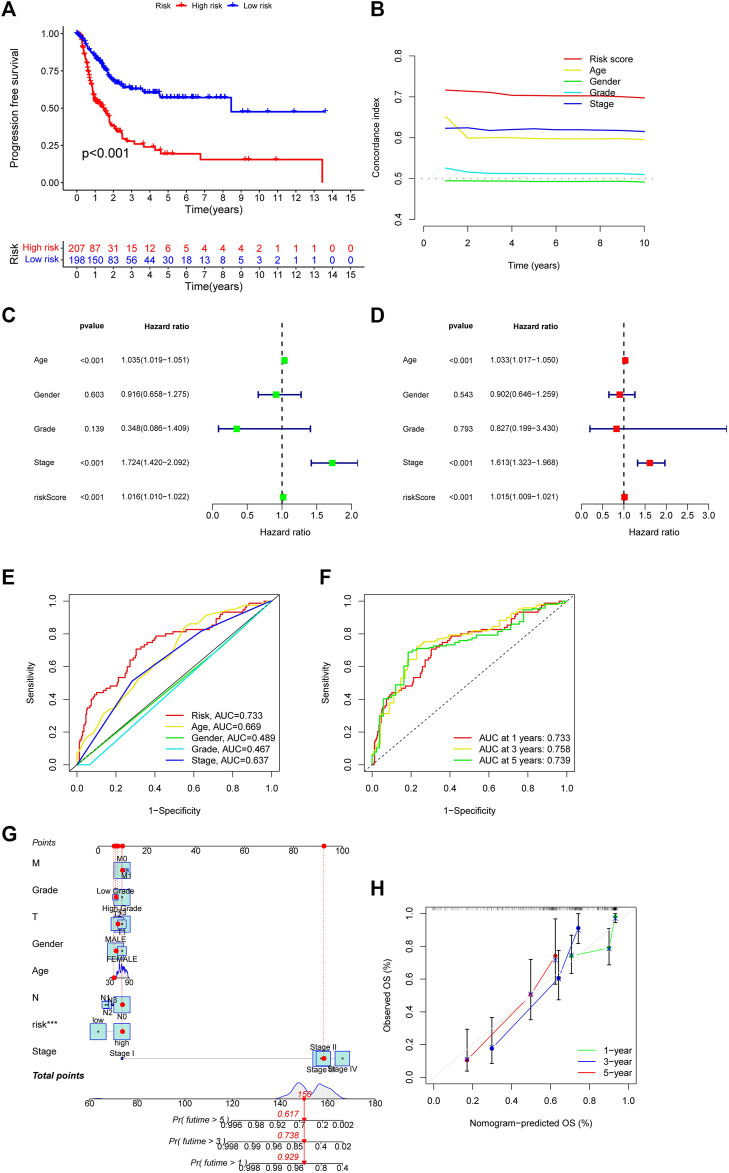
Evaluation of CupRLSig model predictive accuracy using the all TCGA-BLCA group **(A)** Kaplan–Meier curves for progression-free survival of BLCA patients **(B)** C-index curve of the risk model depicts risk scores and other clinical parameters that predict BLCA patients’ prognosis **(C)** Forest plots for univariate Cox proportional-hazard analysis **(D)** Forest plots for multivariate Cox proportional-hazard analysis **(E)** ROC curve of the CupRLSig risk score and other clinicopathological variables **(F)** Time-dependent ROC curves for 1-, 3-, and 5-year survival for the CupRLSig **(G)** A nomogram combining clinicopathological parameters and risk scores predicts 1-, 3-, and 5- year survival probabilities of BLCA patients **(H)** Calibration curves assess the agreement between actual and predicted overall survival at 1-, 3-, and 5- years.

Then, we used the download from http://xena.ucsc.edu/to evaluate the prediction accuracy of our CupRLSig prediction model in BLCA patients. From our results, PFS was significantly shortened in high-risk patients (Figure 4A, *p* < 0.001), and C-index shows that the model’s discrimination is most evident (Figure 4B). By univariate and multivariate Cox regression analysis, the results showed that the CupRLSig risk score could be used as an independent prognostic element (Figures 4C, D). Compared with other clinicopathological variables, its AUC of 0.733 is a better predictor of the prognosis of BLCA (Figure 4E). The AUC at 1, 3 and 5 years were 0.733, 0.758 and 0.739 under the constructed model. This could be considered that CupRLSig had a satisfactory prognosis (Figure 4F). The CupRLSig risk score combined with staging, gender, age and other factors, a nomogram was developed to guide the clinical prognosis evaluation and estimate the 1-year, 3-year and 5-year survival probabilities of patients with BLCA (Figure 4G). As shown in Figure 4G, the corresponding score of BLCA patients is 150. So the 5, 3 and 1-year survival rate was 0.617, 0.738, and 0.929, respectively. The plotted calibration curve is close to the diagonal line, indicating that the constructed model has high accuracy in predicting the survival of patients (Figure 4H).

**FIGURE 5:**
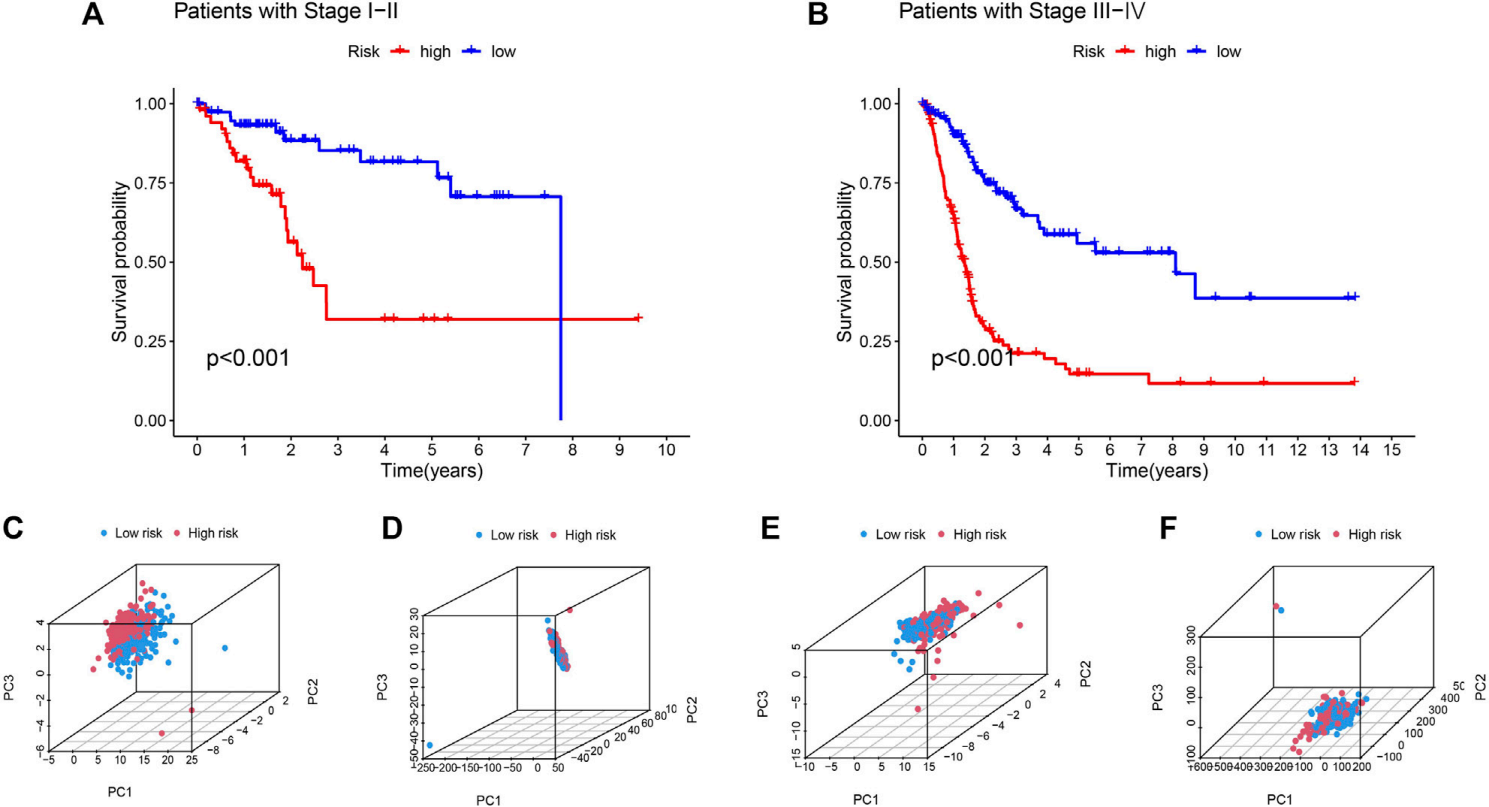
Kaplan-Meier survival curves by clinicopathological variables and PCA of different gene sets performed for patient risk classification. Stage **(A)** Ⅰ-Ⅱ, **(B)** Ⅲ-Ⅳ. PCA of low- and high-risk groups based on **(C)** CupRLSig model risk lncRNAs, **(D)** cuproptosis-related lncRNAs, **(E)** cuproptosis-related genes, and **(F)** all-genes. Red represents patients with high-risk scores and blue represents patients with low-risk scores.

Based on their clinical stage, the patients were divided into two groups, including low-risk group (I-II) and high-risk group (III-IV) to verify the clinical grouping model. The results showed that the model was applied to not only early patients (Figure 5A, *p* < 0.001) but also late patients (Figure 5B, *p* < 0.001). PCA was performed on risk lncRNAs, cuproptosis lncRNAs, cuproptosis genes and whole genes in the CupRLSig model to distinguish high-risk patients from low-risk patients (Figures 5C–F). The study found that the risk lncRNA model (Figure 5C) can effectively differentiate low-risk and high-risk groups, highlighting the model’s accuracy.

### 3.4 Functional enrichment analysis of lncRNAs

**FIGURE 6:**
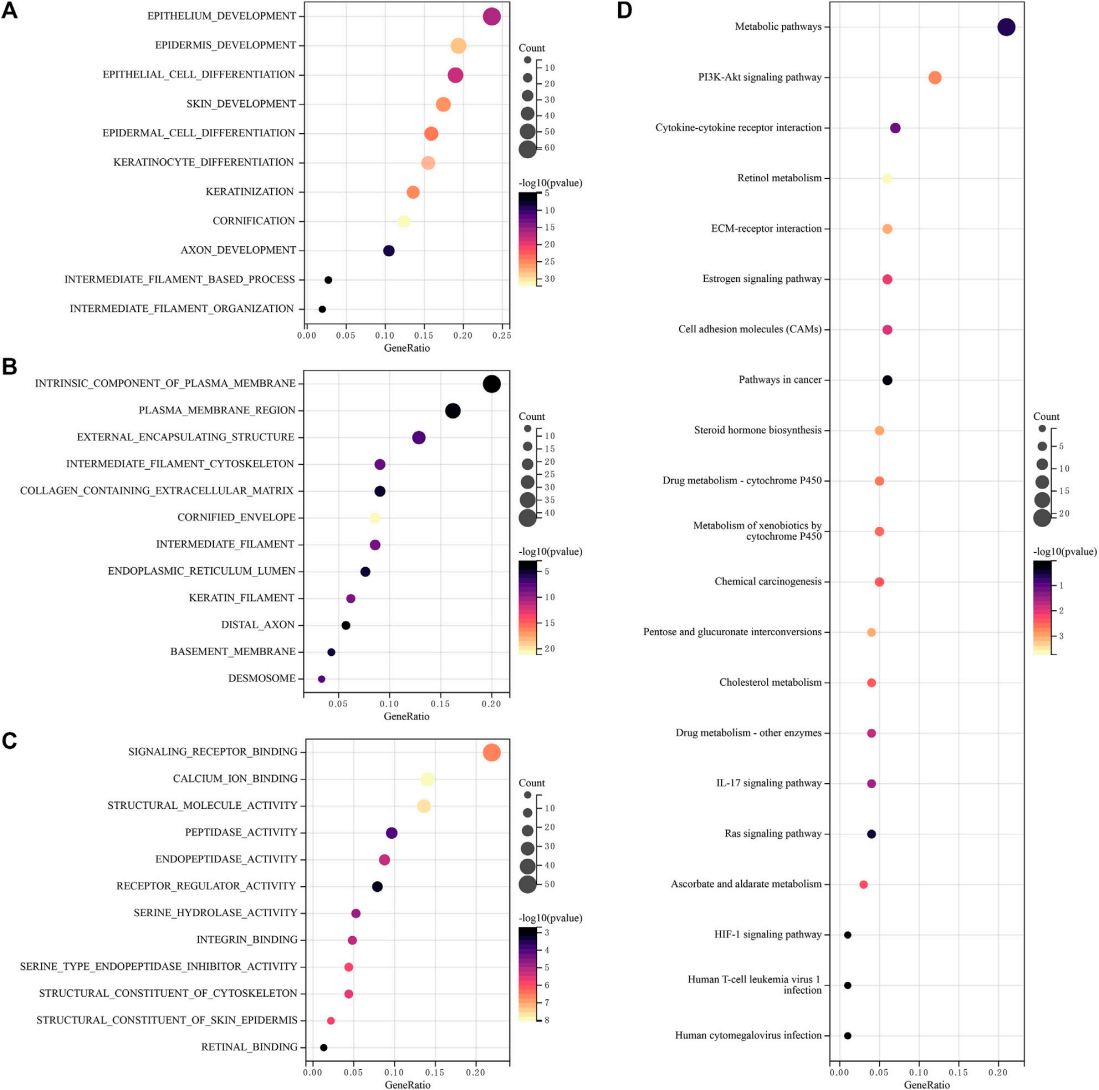
Functional enrichment analysis of lncRNAs **(A–C)** Differentially expressed genes and lncRNAs were enriched in biological process GO terms **(A)** BP (biological pathway), **(B)** CC (cytological component), **(C)** MF (molecular function), **(D)** Differentially expressed genes and lncRNAs were enriched in the KEGG pathways. GO, gene ontology, KEGG, Kyoto encyclopedia of genes and genomes.

To analyze the function of lncRNAs, the related pathways were analyzed using GO and KEGG databases. GO included three sorts: BP (biological pathway), CC (cytological component), and MF (molecular function). These lncRNAs chiefly participated in epithelium development, epidermis development, epithelial cell differentiation, an intrinsic component of plasma membrane, plasma membrane region, cornified envelope, intermediate filaments, endopeptidase activity, signaling receptor binding, calcium ion binding, structural molecule activity and so on (Figures 6A–C) in GO analysis. In addition, these differential lncRNAs chiefly participated in metabolic pathways, PI3K-Akt signaling pathway, cytokine-cytokine receptor interaction, cell adhesion molecules (CAMs) and retinol metabolism in KEGG pathway analysis (Figure 6D).

### 3.5 Immune-related function analysis and gene mutation frequency comparison

**FIGURE 7:**
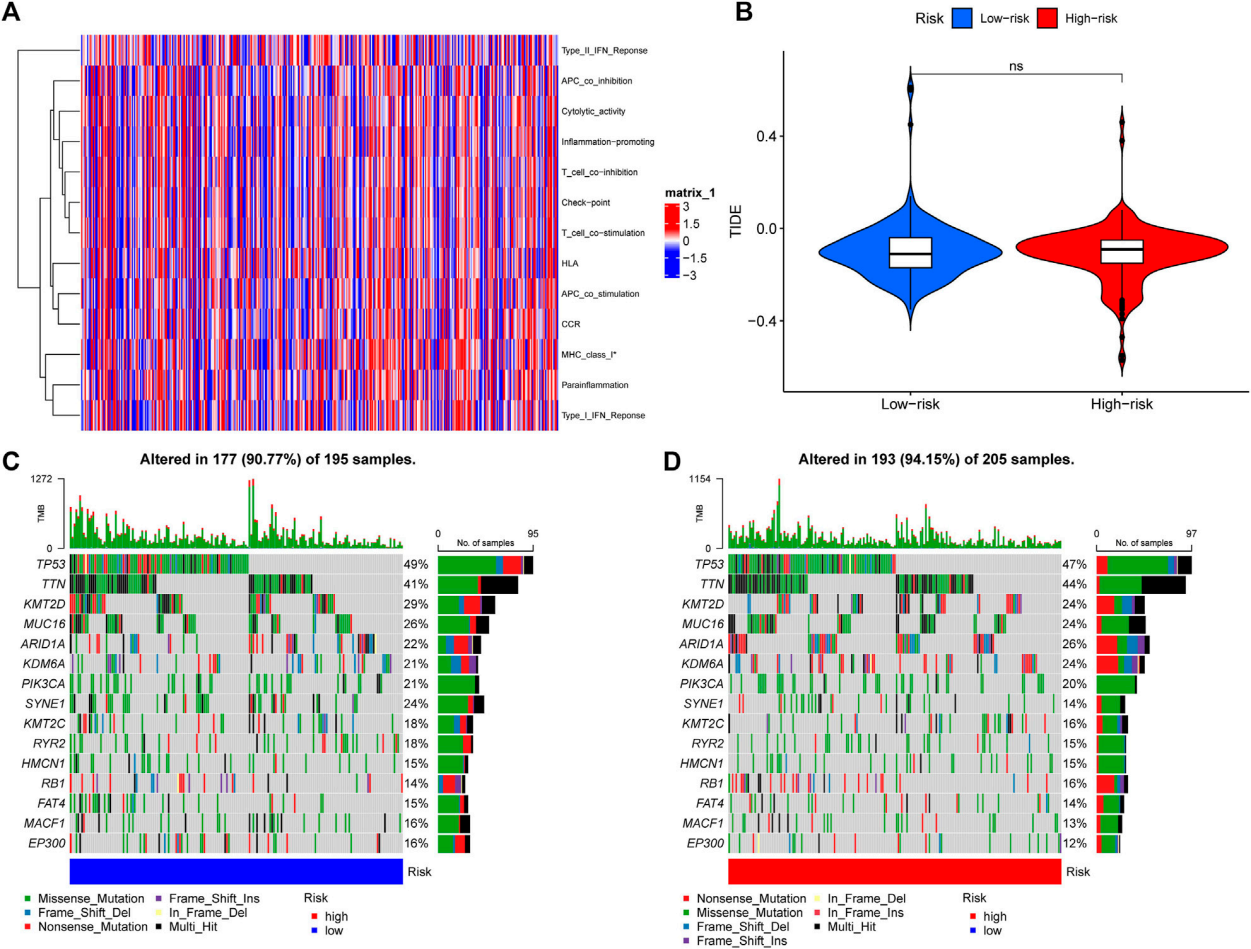
Immune-related function analysis and gene mutation frequency comparison **(A)** The heatmap shows prominent differences in 13 immune-related functions between high- and low-risk groups. *p < 0.05 **(B)** TIDE between high and low-risk groups, TIDE, tumor immune dysfunction and exclusion. Ns means not significant. The waterfall plots show somatic mutations of the most obvious 15 genes between low-risk **(C)** and high-risk **(D)** BLCA patients.

Corresponding analyses were performed to understand the differences in immune-related functions between the high-risk and low-risk groups. Only one immune-related function of MHC-class-I differed between the high-risk and low-risk groups (Figure 7A). The effect of tumor immune dysfunction and exclusion (TIDE) in high-risk and low-risk patients was assessed with no significant difference (Figure 7B). In addition, the mutation frequencies of TTN, ARID1A, KDM6A, and RB1 genes were low in the low-risk group, on the contrary, the mutation frequencies of TP53, KMT2D, MUC16, KDM6A, PIK3CA, SYNE1, KMT2C, RYR2, EP300, MACF1, and FAT4 genes were significantly high in the low-risk group (Figures 7C, D).

### 3.6 Tumor mutation burden and survival analysis

**FIGURE 8:**
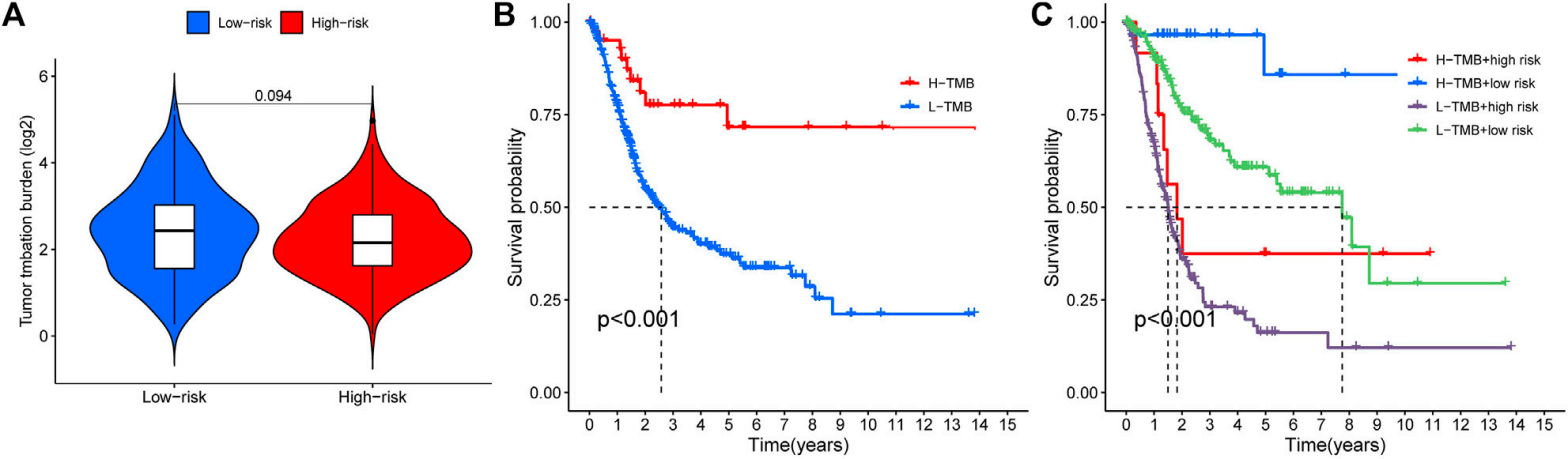
Tumor mutation burden and survival analysis **(A)** TMB comparison between low- and high-risk groups **(B)** Kaplan-Meier curves for high- and low-TMB groups **(C)** Subgroup analyses for Kaplan-Meier curves of patients stratified by TMB and risk scores. *means *p* < 0.05, **means *p* < 0.01, ***means *p* < 0.001.

We analyzed any differences in TMB between high-risk and low-risk patients. As can be seen, there was no significant difference in TMB between the high-risk and low-risk groups (Figure 8A, *p* = 0.094). We found a remarkable difference in the survival analysis of TMB between the high-risk and low-risk groups (Figure 8B, *p* < 0.001), as well as in the combined analysis of TMB and patient risk (Figure 8C, *p* < 0.001).

### 3.7 High-mutation genes are associated with tumor immune infiltration in BLCA

**FIGURE 9:**
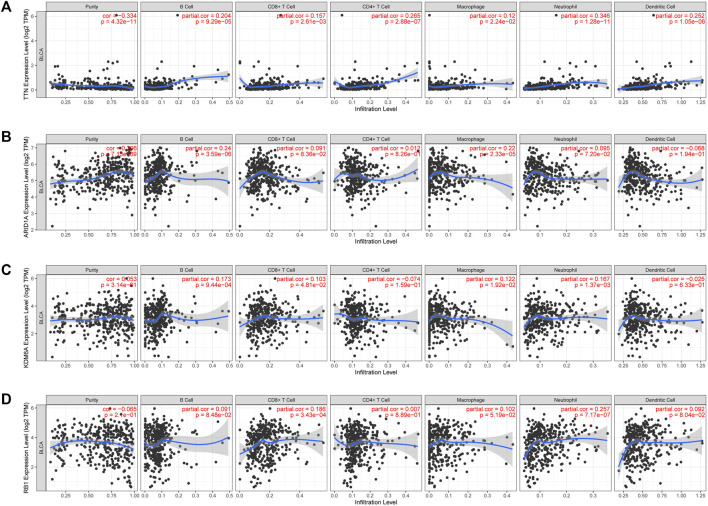
The association between four high-mutation genes and immune infiltration. The relevance between the expression of TTN **(A)**, ARAD1A **(B)**, KDM6A **(C)**, and RB1 **(D)** and the abundance of immune cells in BLCA.

Cuproptosis plays a crucial role in developing the tumor’s immune microenvironment. In our study, we also elucidated the association of the four genes (TTN, ARID1A, KDM6A, RB1) above with high mutation frequency in the high-risk group with immune infiltration of BLCA by the TIMER database. Our findings revealed that TTN expression was positively associated with the abundance of B cells (*p* = 9.29e-05), CD8^+^ T cells (*p* = 2.61e-03), CD4^+^ T cells (*p* = 2.88e-07), neutrophils (*p* = 1.28e-11), macrophages (*p* = 2.24e-02), and myeloid dendritic cells (*p* = 1.05e-06) (Figure 9A); the correlation between ARID1A expression and the abundance of B cells (*p* = 3.59e-06) and macrophages (*p* = 2.33e-05) is also positive, but not significant association with CD8^+^ T cells (*p* = 8.36e-02), CD4^+^ T cells (*p* = 8.26e-01), neutrophils (*p* = 7.20e-02) (Figure 9B). However, there was a negative association between KDM6A expression and the abundance of B cells (*p* = 9.44e-04), CD8^+^ T cells (*p* = 4.81e-02), neutrophils (*p* = 1.37e-03), and macrophages (*p* = 1.92e-02), but no association with the expression of CD4^+^ T cells (*p* = 1.59e-01) and myeloid dendritic cells (*p* = 6.33e-01) (Figure 9C); a positive relevance between RB1 expression and the abundance of CD8^+^ T cells (*p* = 3.43e-04), neutrophils (*p* = 7.17e-07) was described, but no association with the abundance of B cells (*p* = 8.48e-02), CD4^+^ T cells (*p* = 8.89e-01), macrophages (*p* = 5.19e-02), and medullary dendritic cells (*p* = 8.04e-02) (Figure 9D).

### 3.8 High mutation genes are associated with drug sensitivity and immune checkpoint analysis in BLCA

**FIGURE 10:**
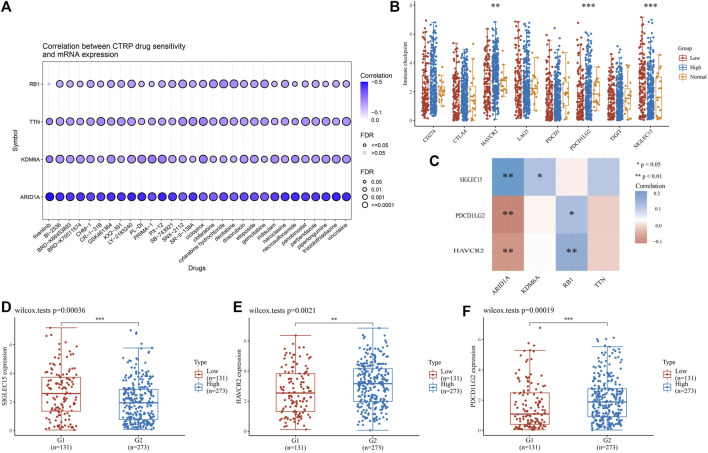
High-mutation genes are associated with drug sensitivity and immune checkpoints analysis **(A)** Drug-sensitivity analysis of four high-mutation genes in BLCA **(B)** Different expressions of immune-checkpoints in low-, high-grade and normal adjacent tissues in BLCA **(C)** The correlation analysis between four high-mutation genes and three immune-checkpoints genes **(D–F)** The expression of three immune-checkpoints genes in low- and high-grade in BLCA **(D)** SIGLEC15, **(E)** HAVCR2, **(F)** PDCD1LG2 (*p < 0.05, **p < 0.01, ***p < 0.001, asterisks (*) stand for significance levels.).

Chemotherapy is essential in the course of cancer treatment. Therefore, the mode of cancer treatment can be more precisely known by drug sensitivity analysis, and our results showed that the potential of TTN, ARID1A, KDM6A, and RB1 as drug scanning targets for BLCA, indicating that the expression of TTN, ARID1A, KDM6Aand RB1 is positively associated with most drugs in CTRP (Figure 10A). Later, BLCA samples were divided into high-risk and low-risk groups to perform immune checkpoint analysis with normal adjacent tissues. The expression of HAVCR2, PDCD1LG2 and SIGLEC15 is prominently different in normal and tumor samples (Figure 10B). After that, we proceeded with an association analysis between four highly mutated genes and immune checkpoint-related genes. We found that ARID1A was significantly positively correlated with SIGLEC15, KDM6A was significantly positively correlated with SIGLEC15, RB1 was significantly positively correlated with HAVCR2 and PDCD1LG2, while ARID1A was negatively correlated with HAVCR2 and PDCD1LG2 (Figure 10C). Finally, we performed gene expression analysis of these three immune checkpoint genes in low-risk and high-risk groups of BLCA and showed significant differences in the expression of SIGLEC15 (Figure 10D, *p* = 0.00036), HAVCR2 (Figure 10E, *p* = 0.0021), and PDCD1LG2 (Figure 10F, *p* = 0.00019) genes.

## 4 Discussion

Increasing evidence suggests a role for cuproptosis in prognostic cancer evaluation (Bian et al., 2022), but the prognostic value of CRGs in BLCA is unknown. With the deepening of lncRNA research, the mechanism of lncRNAs in BLCA has been elucidated and found some lncRNAs may become novel diagnostic and prognostic markers for BLCA (Quan et al., 2018). Our study further used bioinformatics methods to elucidate the relationship between CRGs, lncRNAs, and prognostic value in BLCA. Our results suggested that cuproptosis-related lncRNAs and CRGs have prognostic value in BLCA, providing a corresponding basis for the following study.

Gene expression profiling showed that SLC31A1, LIPT2, CDKN2A, and GCSH expression was increased in tumor tissues, while NFE2L2, NLRP3, ATP7A, DLD, MTF1, and DLST expression was decreased in tumor tissues compared with normal tissues. It has previously been shown that patients with multiple duplications at the CDKN2A/ARF locus have poor survival, suggesting that multiple duplications, in combination with other genetic changes, have synergistic effects and negatively impact on BLCA prognosis (Berggren de Verdier et al., 2006); NLRP3 is the best-studied member of the NLR family, and it plays a crucial role in a variety of inflammatory pathologies (Inouye et al., 2018), and in an early tissue survey of NLRP3 expression, Kummer et al. found the presence of NLRP3 in the human bladder (Kummer et al., 2007). Among the lncRNAs investigating CRGs, a total of 15 CRGs were explored to co-express with BLCA lncRNAs, and the heatmap of the association between CRGs and cuproptosis-related lncRNAs also showed that most of them were positively correlated. The high and low-risk expression of lncRNAs in BLCA distinguished 29 low-risk lncRNAs and 22 high-risk lncRNAs. Numerous kinds of research have found that lncRNAs play a crucial role in the development of BLCA. Previous studies have also demonstrated six lncRNAs (LINC02195, LINC01484, LINC01468, SMC2-AS1, AC011298.1, and PTPRD-AS1) are of great value in the prognosis of BLCA (Gao et al., 2019). Wang et al. (Wang et al., 2019) found that LINC01296 is overexpressed in BLCA, high expression of LINC01296 promotes the proliferation of BLCA cells, and high expression of LINC01296 in BLCA is significantly associated with poor prognosis of BLCA. However, the study of lncRNAs expressed in BLCA is largely unclear. Therefore, in this study, we constructed a prognostic model by cuproptosis-related lncRNAs and predicted the association between lncRNAs and BLCA. The constructed model was found to be of significant value in predicting the prognosis of BLCA.

In GO enrichment analysis, these lncRNAs were mainly involved in epithelium development, epidermis development, epithelial cell differentiation, an intrinsic component of plasma membrane, plasma membrane region, cornified envelope, signaling receptor binding, intermediate filaments, calcium ion binding, endopeptidase activity, structural molecule activity and other functions. Previous studies have demonstrated a decrease in epidermal development activity in bladder luminal tumors (Trilla-Fuertes et al., 2019), which can be a target for neoadjuvant therapy of BLCA. Immunohistochemistry of intermediate filaments (IF) is an important method to assess tumor epithelial, mesenchymal, muscle, glial, or neural differentiation (Miettinen et al., 1984). At the same time, lncRNAs are involved in the function of intermediate filaments, which reflects that lncRNAs can also indirectly assess tumor epithelium and have important reference value for tumor diagnosis, treatment, and prognosis, endopeptidase activity is different in the expression of normal tissues and tumor groups, and in previous studies, it has been found that cysteine endopeptidase activity is increased in colorectal adenomas. There are also differences in activity at different stages of tumors (Shuja et al., 1991). In KEGG enrichment analysis, differential lncRNAs are mainly involved in the PI3K-Akt signaling pathway, retinol metabolism function. Phosphoinositide 3′-kinase (PI3K)/Akt signaling cascades control cellular processes, such as apoptosis and proliferation. In addition, it is a mediator of insulin action on target cells and a major modulator of energy metabolism (Barthel et al., 2007). Studies found that men with higher serum retinol levels have a lower risk of developing advanced hepatocellular carcinoma than men in the lowest quartile (Yuan et al., 2006). That is, serum retinol expression levels are associated with the risk of cancer, and similarly, in BLCA, retinol expression levels are associated with the development of BLCA. At the same, vitamin A is a precursor of retinol, and in tumor tissues, vitamin A expression levels are decreased. Therefore, vitamin A administration may be beneficial in preventing and treating human BLCA (Yalçin et al., 2004). BLCA can promote changes in the bladder wall, thereby affecting the regulation of cell adhesion molecules (CAMs) and affecting the integrity of the tissue (Pontes-Júnior et al., 2013; Wu et al., 2017).

In immune-related function analysis, only one immune-related function of MHC-class-I differs, major histocompatibility complex (MHC) class I and II products play a central role in immune response function by limiting T cell recognition of foreign antigens (Cabrera et al., 2003), and downregulation or loss of expression of one or more human leukocyte antigen (HLA) alleles by any mechanism can reduce polymorphisms, thereby reducing the ability to present antigens through MHC products.

Four genes with high mutation frequency (TTN, ARID1A, KDM6A, RB1) were identified by gene mutation frequency comparison, and immune infiltration, drug sensitivity and immune checkpoint analysis were performed for these four genes. In immune infiltration analysis, these four genes showed a significant association with most immune cells, and in previous studies, TTN is a frequently mutated gene in BLCA and can be used as a biomarker to predict immune response (Zhu et al., 2020); ARID1A is a component of the SWIth/Sucrose Non-Fermentable (SWI/SNF) chromatin remodeling complex, and its function is to control many important biological processes, such as tumor microenvironment regulation and anticancer immunity (Conde and Frew, 2022; Lu et al., 2022), presenting a significant association with the expression of immune cells. KDM6A is a chromatin-regulated gene with the highest mutation frequency in muscle-invasive BLCA and non-muscle-invasive BLCA cohorts. More than half of KDM6A mutations result in shorter proteins (Kim et al., 2022), which impairs protein expression and decreases immune function. In drug sensitivity analysis, we found that low expression of these four genes (TTN, ARID1A, KDM6A, and RB1) was positively associated with resistance to cancer therapeutics response portal (CTRP). BI2536 is a Polo-like kinase 1 (PLK1) inhibitor with *in vitro* antitumor activity against BLCA cell lines RT4, 5637, and T24, preventing cell proliferation and clonogenicity, thereby significantly inhibiting BLCA growth and spread (Brassesco et al., 2013). Clofarabine is an anti-metabolic drug used as a third-line treatment for children with acute lymphoblastic leukemia. In an *in vitro* study, clofarabine was also shown to be effective in remission of BLCA patients (Ertl et al., 2022). Similarly, drugs such as ciclopirox, cytarabine hydrochloride, etoposide have been reported to be effective in the treatment of BLCA (Weir et al., 2021; Kapur et al., 2022; Reyhanoglu and Tadi, 2022). However, drugs as indisulam, necrosulfonamide, triazolothiadiazine are firstly proved to be correlated with BLCA risk genes in this paper, which has certain value for future research. In addition, some natural medicines (curcumin, resveratrol, genistein, quercetin, paclitaxel, and silibinin) can also play a role in the treatment of cancer through their role in regulating lncRNAs in cancer through Hedgehog and Hippo signaling pathway components (Sharma et al., 2022). In immune checkpoint analysis, three immune checkpoint genes (HAVCR2, PDCD1LG2, SIGLEC15) were significantly different between high and low-risk groups of BLCA and normal adjacent tissues. Methylenetetrahydrofolate cyclohydrolase (MTHFD2) is a potential oncogene because it is closely associated with poor prognosis and high levels of immune infiltration in BLCA (Zhu et al., 2022), while the expression of HAVCR2 is closely related to MTHFD2. Therefore, the expression of HAVCR2 is valuable in evaluating the prognosis of BLCA. PDCD1LG2, also known as PD-L2, has been previously elevated in Epstein-Barr virus (EBV)-positive tumors (Cancer Genome Atlas Research Network, 2014), but its expression in BLCA is unclear, and in our study, PDCD1LG2 expression was found to be decreased in early-stage patients and essentially flat in advanced patients compared with normal adjacent tissues.

However, our study has a number of shortcomings. Because all our analyzes were performed using the TCGA-BLCA cohort and are best validated in conjunction with the GEO cohort, some of which contain detailed patient characteristics and outcomes. Secondly, due to our limited time, there may be other CRGs included in other literatures, but these genes were not included in our article for study. For immune infiltration, drug sensitivity, and immune checkpoint analyses, we selected only four high mutation risk genes for analysis, while ignoring other potentially valuable genes. In addition, some mechanistic studies should be established to further validate our results.

## 5 Conclusion

Our research identified a total of 21 cuproptosis-related lncRNAs and their survival prognostic features in BLCA, and the results indicated that they could more accurately predict the prognosis of BLCA patients. Afterward, we proceeded with a preliminary analysis of the function of cuproptosis-related lncRNAs. Among immune-related function analysis, only MHC-class-I was significantly different. We found a remarkable difference in the survival analysis of TMB between the high-risk and low-risk groups and high-TBM patients had better OS. Finally, we performed immune infiltration, immune checkpoint and other related functional analysis of four genes (TTN, ARID1A, KDM6A, RB1) that were highly mutated in the high-risk group, and the findings showed that the four genes were significantly relevant to immunity in BLCA. To summarize, this study systematically investigated the prognostic and immune relevance of CRGs and cuproptosis-related lncRNAs in BLCA. These results have certain reference values for the treatment of BLCA.

## Data Availability

The original contributions presented in the study are included in the article/Supplementary Material, further inquiries can be directed to the corresponding author.
