# Supplementary material for: A novel cuproptosis-related lncRNAs signature predicts prognostic and immune of bladder urothelial carcinoma
Source: Front Genet. 2023 Mar 31;14:1148430. doi: 10.3389/fgene.2023.1148430 (PMC10102384; doi:10.3389/fgene.2023.1148430)
Supplement: Supplementary file 1 [file Table1.DOCX]

Supplementary Material

**Supplementary Table S1** The clinical characteristics of BLCA patients in the TCGA cohort.

| Clinical characters | Number |
| --- | --- |
| Gender  Male  Female | 106  300 |
| Age |  |
| Mean (SD) | 68.1 (10.6) |
| Median [MIN, MAX] | 69 [34,90] |
| TNM stage  I  II  III  IV | 2  129  140  133 |
| pT_stage  T1  T2  T3  T4  TX | 11  190  157  42  6 |
| pN_stage  N0  N1  N2  N3  NX | 236  46  75  7  36 |
| pM_stage  M0  M1  MX | 195  11  197 |

**Supplementary Table 2** 19 cuproptosis-related genes

| Symbol | Gene name |
| --- | --- |
| NLRP3 | NLR family pyrin domain containing |
| ATP7B | ATPase copper transporting beta |
| ATP7A | ATPase copper transporting alpha |
| SLC31A1 | Solute carrier family 31 member 1 |
| FDX1 | Ferredoxin 1 |
| LIAS | Lipoic acid synthetase |
| LIPT1 | Lipoyltransferase 1 |
| LIPT2 | Lipoyl(octanoyl) transferase 2 |
| DLD | Dihydrolipoamide dehydrogenase |
| DLAT | Dihydrolipoamide S-acetyltransferase |
| PDHA1 | Pyruvate dehydrogenase E1 subunit alpha 1 |
| PDHB | Pyruvate dehydrogenase E1 subunit beta |
| MTF1 | Metal regulatory transcription factor 1 |
| GLS | Glutaminase |
| CDKN2A | Cyclin dependent kinase inhibitor 2A |
| DBT | Dihydrolipoamide branched chain transacylase E2 |
| GCSH | Glycine cleavage system protein H |
| DLST | Dihydrolipoamide S-succinyltransferase |
| NFE2L2 | NFE2 like bZIP transcription factor 2 |

**Supplementary Table S3** The CupRLSig risk score in BLCA.

| id | coef |
| --- | --- |
| AC131025.3 | 1.61952211349396 |
| LINC01572 | 0.852763833913854 |
| AC002401.1 | 1.85127923980961 |
| AL390236.1 | 1.26804732118412 |
| AC103746.1 | 0.646643474193456 |
| KCNIP2-AS1 | 1.40682848502958 |
| AC055811.3 | 2.05768394915157 |
| AC087289.1 | 1.59493834085492 |
| AL132800.1 | 0.566462909300765 |
| AC022405.1 | 1.4120012861056 |
| AC025442.2 | -1.11578915127572 |
| AC007993.3 | -0.291372257316192 |
| GUSBP11 | 0.585768026214916 |
| AC009088.1 | -1.08427504951988 |
| LINC-PINT | -1.39715258364629 |
| AC124248.2 | -3.1975521678791 |
| AC084781.1 | -0.537175175817353 |
| AC034102.8 | -1.83962616253994 |
| LINCADL | 0.934253441787245 |
| AC092821.3 | -2.41472279290379 |
| JARID2-AS1 | -0.465662085213937 |
